# Integrons in the Age of Antibiotic Resistance: Evolution, Mechanisms, and Environmental Implications: A Review

**DOI:** 10.3390/microorganisms12122579

**Published:** 2024-12-13

**Authors:** Niyaz Ali, Izhar Ali, Ahmad Ud Din, Kashif Akhtar, Bing He, Ronghui Wen

**Affiliations:** 1 State Key Laboratory for Conservation and Utilization of Subtropical Agro-Bio-Resources, College of Life Science and Technology, Guangxi University, 100 Daxue Road, Nanning 530004, China; niyazali200@gmail.com (N.A.); izharali48@gmail.com (I.A.); kashif@zju.edu.cn (K.A.); 2 Guangxi Baise Modern Agriculture Technology Research and Extension Center, Management Committee of Baise National Agricultural Science and Technology Zone of Guangxi, Baise 530108, China; 3 Plants for Human Health Institute, Department of Food, Bioprocessing and Nutrition Sciences, North Carolina State University, Kannapolis, NC 28081, USA; ahmadndwa@yahoo.com; 4 Guangxi Key Laboratory of Agro-Environment and Agric-Products Safety, College of Agriculture, Guangxi University, Nanning 530004, China; hebing@gxu.edu.cn

**Keywords:** integrons, gene cassettes, environment, antimicrobial resistance

## Abstract

Integrons, which are genetic components commonly found in bacteria, possess the remarkable capacity to capture gene cassettes, incorporate them into their structure, and thereby contribute to an increase in genomic complexity and phenotypic diversity. This adaptive mechanism allows integrons to play a significant role in acquiring, expressing, and spreading antibiotic resistance genes in the modern age. To assess the current challenges posed by integrons, it is necessary to have a thorough understanding of their characteristics. This review aims to elucidate the structure and evolutionary history of integrons, highlighting how the use of antibiotics has led to the preferential selection of integrons in various environments. Additionally, it explores their current involvement in antibiotic resistance and their dissemination across diverse settings, while considering potential transmission factors and routes. This review delves into the arrangement of gene cassettes within integrons, their ability to rearrange, the mechanisms governing their expression, and the process of excision. Furthermore, this study examines the presence of clinically relevant integrons in a wide range of environmental sources, shedding light on how anthropogenic influences contribute to their propagation into the environment.

## 1. Introduction

Integrons are genetic elements predominantly found in Gram-negative bacteria, capable of acquiring and expressing open reading frames (ORFs) embedded in gene cassettes, thereby converting them into functional genes [1]. They were first identified in Gram-negative bacteria for their ability to assemble and express diverse antibiotic resistance gene cassettes acquired from the environment [2]. The first case of bacteria exhibiting resistance to multiple antibiotics was identified in Japan in the 1950s [3,4]. However, until the 1970s, it was unclear whether these phenotypes associated with plasmids, particularly with transposable segments within plasmids [5,6]. By the late 1980s, these elements were identified and characterized as genetic structures responsible for capturing and expressing resistance genes, now known as integrons [7]. It is now well established that these mobile gene cassettes are major carriers of antimicrobial resistance genes (ARGs) in most Gram-negative bacteria, and, to some extent, in Gram-positive bacteria, too [8,9]. Their prevalence and evolution have been extensively studied in epidemiological research, particularly in clinical and agricultural environments, underscoring their significance. In the late 1990s, chromosomal integrons were discovered in bacteria with no apparent role in resistance. This finding coincided with the identification of super integrons in the genome of *Vibrio Cholera* and similar fragments in environmental bacterial populations [9,10]. The evolutionary history of chromosomal integrons suggests that this adaptive genetic system has helped Gram-negative bacteria to adapt to environment changes [11,12]. Furthermore, the evolutionary relationships and mechanisms of chromosomal integrons play a critical role in the emergence of multiple antibiotic resistance [13,14].

In the present era, antimicrobial resistance (AMR) has become a critical global health concern, and the transfer of resistance genes and pathogens among humans, animals, and the environment has reached alarming levels. This interconnected spread underscores the urgent need for comprehensive strategies to manage and mitigate resistance across multiple sectors [15]. Despite the existence of natural and anthropogenic barriers that limit gene and bacterial flow, pathogens and other bacteria continue to acquire resistance genes from other species and environmental sources. This exchange significantly hampers efforts to prevent and treat bacterial infections [16]. The increased prevalence of ARGs and resistant bacteria in the environment is largely driven by selective pressures imposed by human activities. This selective pressure arises from the overuse of antimicrobial agents (AMA) in hospitals, industry, poultry farming, and agriculture, as well as practices like the use of antibiotics to prevent diseases and promote growth in farm animals and aquaculture. The emergence of antimicrobial resistance (AMR) is a complex evolutionary process, with integrons playing a pivotal role. Integrons act as key facilitators in bacterial evolution and the dissemination of resistance genes. This review discusses the structure and evolution of integrons, their role in AMR, and the distribution of resistance gene cassettes across various environments, shedding light on their critical contribution to the global AMR crisis.

## 2. Integrons Structure, *Function* and Evolution

### 2.1. The Mechanism of Integron Functionality: Acquisition, Incorporation, and Expression of Gene Cassettes

**Figure 1:**
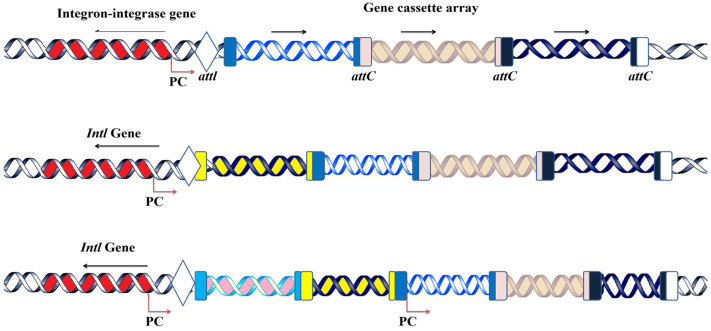
Integrons structure, acquisition and functions. Gene cassettes are sequentially inserted into an array by recombination between *attI* and the cassette associated-recombination sites, *attC*; Pc, an integron-carried promoter; and *intI*, a gene for the integron integrase. A single ORF (arrow) is expressed by the Pc promoter on gene cassettes (various genes are depcited with various colors). Pc is located between *intI* and *attI* in certain integrons. Cassettes have two ORFs, no ORF, or an ORF pointing in the opposite direction. *IntI* is transcribed in the same direction as the gene cassettes in certain genera.

The primary characteristics of integrons include the following three core features: (i) acquiring gene cassettes from external sources, (ii) incorporating these genes into their structure, and (iii) subsequently expressing them as part of their genetic framework [2,17]. A critical component of integrons is the *Intl* gene, which encodes an enzyme known as integrase (Figure 1) [18]. Integrase belongs to the tyrosine recombinase family [19], and it catalyzes site-specific recombination between the *attl* and *attC* sites to integrate these circular gene cassettes into a cassette array [19].

The recombination between the *attI* sites of integrons and the *attC* sites of gene cassettes is initiated by the integron-encoded integrase enzyme [20]. A critical component of integrons is the associated promoter (Pc), which drives the expression of gene cassettes following their insertion [1,18]. The basic structure of a gene cassette includes an open reading frame (ORF) flanked by a recombination site, known as the 59-base element (attC), which facilitates its integration into the integron [21]. Future research should prioritize the exploration and characterization of novel integrons in diverse environmental niches, as their unique structure holds potential beyond antimicrobial resistance (AMR). In addition to their role in AMR, integrons exhibit a distinct architecture that could be leveraged for innovative applications. Modifying and engineering integrase enzymes to enhance their specificity and efficiency may pave the way for advanced biotechnological applications, including the development of customized gene constructs.

### 2.2. Class 1 Integrons’ Gene Cassette Acquisition and Expression System

**Figure 2:**
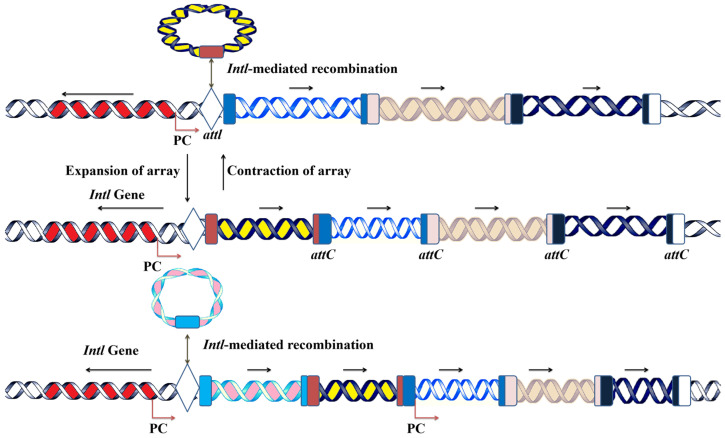
Acquisition of gene cassettes into the integron’s platform. New gene cassettes are acquired through the recombination of the *attl* site of integrons and the *attC* site of the circular gene. The new incoming gene is inserted at the proximal position of the integrase gene next to the embedded promoter. The repeated acquisition leads to the expansion of the cassette array, but the cassettes can be excised in the reverse of acquisition either by *att1-attl* or *attC-attc* recombination.

Gene cassettes typically consist of an attC recombination site and an open reading frame (ORF), requiring an external promoter for their expression. Extensive research has focused on the expression system of class 1 integron cassettes, which is driven by one of two promoters, as follows: *Pc1*, located within the *intI1* gene, or *Pc2*, situated in the *attI* sites [22]. These promoters have been categorized based on their strength [23]. Integrons carrying weak promoters often exhibit higher cassette excision rates, as weaker promoter activity may destabilize the maintenance of gene cassettes within the integron [24,25]. Moreover, the position of a gene cassette relative to the integron’s promoter plays a critical role in its expression levels. Gene cassettes located closer to the integron integrase promoter maintain stable expression, whereas those situated farther away exhibit progressively reduced expression rates (Figure 2) [26,27].

The clinical class 1 integrons rarely contain more than six cassettes, and this may be the reason why the cassettes at a distance from the *Pc1* promoter are often not expressed [28,29]. However, some gene cassettes carry their own promoters, enabling independent expression regardless of their position within the integron array. For example, the chloramphenicol resistance gene cassette (*cmlA*) contains its own promoter [30], as do quinolone resistance genes from the qnrVC family [31], allowing them to maintain expression irrespective of their distance from the integron-associated promoters [32]. It is, therefore, unsurprising that certain cassettes possess independent promoters, ensuring their functionality and evolutionary persistence [33]. Metagenomic analyses have also identified numerous gene cassettes lacking an ORF, suggesting that these ORF-less cassettes may have evolved as mobile promoter cassettes, potentially serving regulatory or auxiliary functions within integrons [30]. Gene cassettes may originate as mobile promoter cassettes [34]. While some gene cassettes have their own promoters, they may also lead to rearrangement within the cassette array to express the desired gene in response to specific stress, utilizing a single promoter.

### 2.3. Phylogenetic Analysis of the IntI Gene and Its Evolution

**Figure 3:**
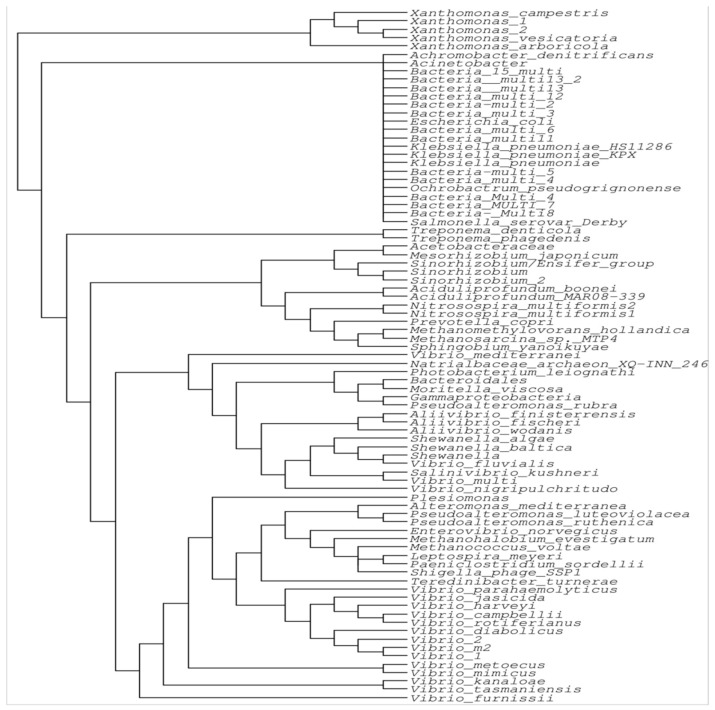
Phylogenetic analysis of the integron integrase gene in bacteria. The phylogenetic tree shows that *Xanthomonas* has appeared as the parental species for the integron integrase gene, and *Vibrio* spp. and Bacteria multi spp. make separate single clade. In contrast, *Aliivibrio Wodan* is up to *Shewanella*, which includes 13 species that make a distinct clade. Three other small clades are also presented in the phylogenetic tree. The analysis shows that the integrons of different clades are more diverse, showing that the amino acid identity is very low and yet to be classified.

Integrons are genetic elements that encode the *IntI* gene [32], which belongs to the tyrosine recombinase family and includes an additional 16-amino-acid motif that significantly enhances its activity [35]. Approximately 15% of bacterial genomes contain integrons, as indicated by the presence of the *Intl* gene [25,36]. Integrons are found in a variety of environmental sediments, including those in hot springs, rivers, seas, soils, plant surfaces, rhizospheres, and Antarctic soils [18,35]. Although the percentage cutoff criteria to discriminate between different classes has not yet been established, hundreds of distinct integron families have been found in the last few decades, which are distinguished based on the homology of the *Intl* gene sequence [25]. We collected sequences of the integrase gene from the NCBI data bank and ran them through Mega6 Software (version 6.06) to obtain their phylogenetic analysis. The results revealed that *Xanthomonas* is the main clade, and the rest of the integrase genes are extended from this clade, which confirms that *Xanthomonas* is the parental strain carrying the integron integrase gene (Figure 3).

## 3. The Role of Integrons and Gene Cassettes in Shaping Antimicrobial Resistance: Clinical Relevance and Bacterial Associations

### 3.1. The Role of Integrons in Shaping the Landscape of AMR

Integrons play a pivotal role in the dissemination of antibiotic resistance, particularly in Gram-negative bacteria. Resistance integrons are often associated with conjugative transposons and plasmids, enabling their transfer between cells and even across species [13]. Integrons are categorized into five classes, all linked to antibiotic resistance. Classes 1, 2, and 3 are commonly identified in clinical settings, while class 4 is associated with the SXT element found in *Vibrio cholerae* [37]. Class 5, in contrast, has been discovered in the pRSV1 plasmid of *Aliivibrio salmonicida*. These integrons harbor a diverse array of gene cassettes, with approximately 130 different resistance genes having been identified, reflecting broad phylogenetic diversity [16]. The cassettes within mobile integrons are typically short, with the longest recorded arrays containing up to eight cassettes [38]. It has been suggested that these arrays are regulated by a single promoter, which may lead to the reduced expression of cassettes positioned further downstream [36].

### 3.2. Clinically Relevant Integrons and Their Gene Cassettes

**Figure 4:**
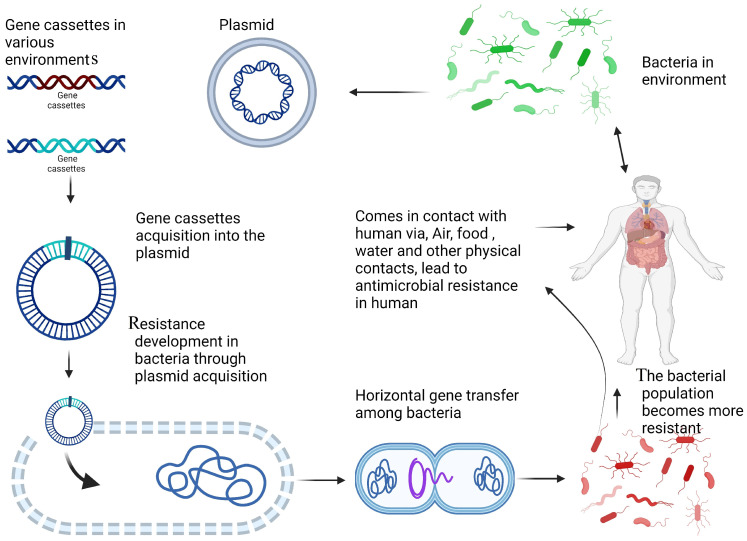
Mechanism of antimicrobial resistance (AMR) development and dissemination. Gene cassettes are present in various environments and can integrate into plasmids within bacteria. The acquisition of these gene cassettes enables bacteria to develop resistance to antimicrobial agents. Environmental bacteria can interact with humans through air, food, water, and physical contact, facilitating the transmission of resistant bacteria. Humans may encounter resistant bacteria, leading to increased AMR in human-associated bacterial populations.

In recent years, the misuse and overuse of antibiotics, coupled with poor management practices, have significantly contributed to the rise in antibiotic resistance. Bacteria develop resistance through the acquisition of resistance genes and genetic mutations. A key factor in the emergence of antibiotic resistance is horizontal gene transfer (HGT), through which bacteria obtain resistance genes via plasmids and transposons [39]. Integrons carry a variety of genes cassettes encoding AMR, with over 130 gene cassettes having been identified, exhibiting different codon patterns and attachment sites [40]. The integrons share common features, ranging from short to long arrays of gene cassettes, and typically accumulate ARGs [19]. These shared characteristics are not inherited from ancestral lineages, but rather arise due to the strong selective pressure exerted by the use of antibiotics and other human activities. Clinically relevant integrons and their gene cassettes play an important role in the dissemination of AMR into the environment (Figure 4) [41].

### 3.3. Clinically Relevant Bacteria and ARG Cassettes

*E. coli* is a noteworthy species within the *Enterobacteriaceae* family, known for carrying integrons and ARGs, and is a common cause of gastrointestinal infections [42]. It contributes to the spread of antibiotic resistance by acquiring multiple resistance genes through various mechanisms, including plasmids, transposons, and integrons [43]. The *E. coli* strains carrying resistance genes through integrons are listed in Table 1. Furthermore, *Acinetobacter baumannii* is a significant source of nosocomial infections and contributes substantially to hospital-acquired infections. *Acinetobacter baumannii* can carry resistance genes, such as *Beta-lactamse*, *metallo-beta-lactamase*, *Amp C*, and class *D beta lactamse* (*carbaphenems*), along with other associated insertion sequences [44].

**Figure 5:**
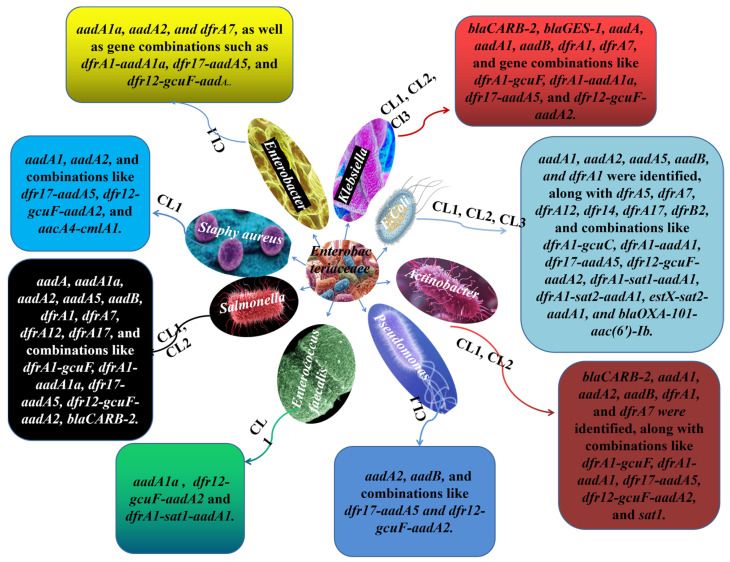
*Enterpbacteriacae* family, members species and their resistance genes. CL is the abbreviation of integrons, and numbers 1, 2, and 3 represent their class. The different boxes extended with arrows represent the various resistance genes carried by that specific bacteria spp. The various colors and shapes present various species extended from the *Enterobacteriacae* family in the middle.

Moreover, *Salmonella* is another resistant pathogenic bacterium, and a key contributor to food-borne illness, usually transmitted through food items such as meat, eggs, and milk. Its capacity for multidrug resistance poses a serious public health threat. *Salmonella* species harbor different classes of integrons, with class 1 integrons being the most prevalent, often carrying multiple resistance genes [45,46]. In addition, *Klebsiella* species are associated with pneumonia, urinary tract infections, and other bloodstream infections. These bacteria exhibit multidrug resistance, and the integrons in *Klebsiella* carry various resistance genes, as listed in Table 1. Furthermore, other species within the *Enterobacteriacae* family, including *Pseudomonas aeroginusa* spp., *Enterococus faecalis* spp., *Enterobacter* spp., and *Staphylococcus* spp., are involved in hospital-acquired infections and exhibit resistance to multiple antibiotics. These bacteria carry a different class of integrons and various ARGs, which have been previously identified (Figure 5) [19].

**Table 1:** Gene cassettes associated with antibiotic resistance and the host.

Gene Cassettes Associated with Antibiotic Resistance	Gene Cassettes	Integron Classes (CL),	Host	References
Erythromycin.	*aadA1*, *aadA2*, *aadA5*, *aadB*, and *dfrA1 were identified*, *along with dfrA5*, *dfrA7*, *dfrA12*, *dfr14*, *dfrA17*, *dfrB2*, and *combinations like dfrA1-gcuC*, *dfrA1-aadA1*, *dfr17-aadA5*, *dfr12-gcuF-aadA2*, *dfrA1-sat1-aadA1*, *dfrA1-sat2-aadA1*, *estX-sat2-aadA1*, and blaOXA-101-aac(6’)-Ib.	CLI, II, III	*Escherichia coli*	[47,48]
Trimethoprim, aminoglycosides, beta-lactamase, and extended spectrum.				
Beta-lactamase enzymes with extended spectrum activity, aminoglycoside antibiotics, and trimethoprim.	*blaCARB-2*, *aadA1*, *aadA2*, *aadB*, *dfrA1*, and *dfrA7* were identified, along with combinations like *dfrA1-gcuF*, *dfrA1-aadA1*, *dfr17-aadA5*, *dfr12-gcuF-aadA2*, and *sat1.*	CLI, II	*Acinetobacter baumannii*	[19]
Aminoglycoside antibiotics, trimethoprim, and extended-spectrum beta-lactamases (ESBLs).	*aadA*, *aadA1a*, *aadA2*, *aadA5*, *aadB*, *dfrA1*, *dfrA7*, *dfrA12*, *dfrA17*, and combinations like *dfrA1-gcuF*, *dfrA1-aadA1a*, *dfr17-aadA5*, *dfr12-gcuF-aadA2*, and *blaCARB-2* were identified.	CLI, II	*Salmonella* spp.	[49,50]
Extended-spectrum beta-lactamases (ESBLs), trimethoprim, and aminoglycoside antibiotics.	*blaCARB-2*, *blaGES-1*, *aadA*, *aadA1*, *aadB*, *dfrA1*, *dfrA7*, and gene combinations like *dfrA1-gcuF*, *dfrA1-aadA1a*, *dfr17-aadA5*, and *dfr12-gcuF-aadA2.*	CLI, II, III	*Klebsiella* spp.	[51,52]
Aminoglycosides and trimethoprim.	*aadA2*, *aadB*, and combinations like *dfr17-aadA5* and *dfr12-gcuF-aadA2.*	CLI	*Pseudomonas aeruginosa*	[29,53]
Trimethoprim, chloramphenicol, and aminoglycosides antibiotics.	*aadA1*, *aadA2*, and combinations like *dfr17-aadA5*, *dfr12-gcuF-aadA2*, and *aacA4-cmlA1*	CLI	*Staphylococcus aureus*	[53]
Trimethoprim and aminoglycosides.	*aadA1a* and gene combinations such as *dfr12-gcuF-aadA2* and *dfrA1-sat1-aadA1.*	CLI	*Enterococcus faecalis*	[54]
Trimethoprim and aminoglycosides.	*aadA1a*, *aadA2*, and *dfrA7*, as well as gene combinations such as *dfrA1-aadA1a*, *dfr17-aadA5*, and *dfr12-gcuF-aadA.*	CLI	*Enterobacter* spp.	[55]

## 4. Environmental Dissemination of Antibiotic Resistance: The Central Role of Integrons Across Ecosystems

### 4.1. The Role of Integrons in the Dissemination of Antibiotic Resistance in the Environment

**Figure 6:**
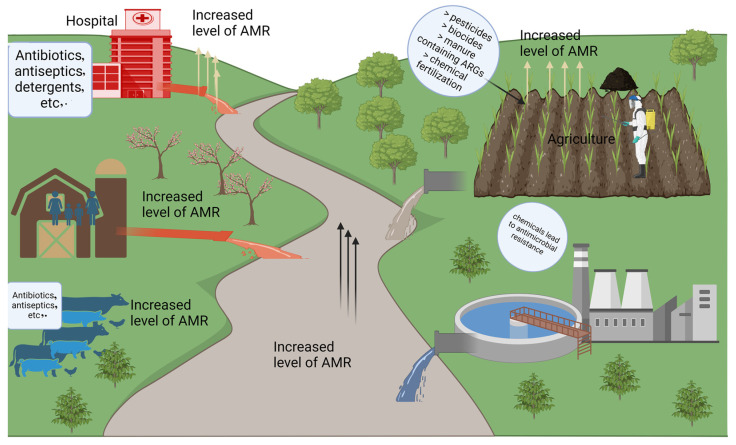
The schematic presentation of sources and pathways of antimicrobial resistance (AMR) in the environment. Anthropogenic activities such as hospital discharge of antibiotics, antiseptics, and detergents; agricultural applications of pesticides, biocides, manure containing antibiotic resistance genes (ARGs), and chemical fertilizers; and livestock farming practices contribute to elevated AMR levels. Wastewater treatment plants and industrial effluents further amplify AMR dissemination, impacting environmental and agricultural ecosystems.

Integrons are present in a variety of environments, from clinical setup to forest soils, aquatic ecosystems, marine sediments, and livestock and agriculture areas. It is believed that these integrons primarily originate from environmental bacteria, and their dissemination and functional role is being influenced by the use of AMA. In response to AMA exposure, bacteria rapidly acquire resistance mechanisms, which contributes to the development of AMR. Different environmental bacteria carry unique gene cassette arrays that are specific to their habitat and conditions [56,57]. These environments may host novel gene cassette arrays, many of which are associated with AMR and are responsive to environmental stressors (Figure 6) [50,58,59].

### 4.2. Integron Integrase Gene as an Effective Proxy for Pollution

Antibiotic resistance poses a significant threat to public and environmental health. The environment is increasingly recognized for its role in spreading resistance, as well as its potential to help mitigate this issue [60]. The widespread and excessive use of medically important antibiotics across agricultural, veterinary, and healthcare fields is a significant contributor to the global rise in AMR [53]. Increasingly, researchers and stakeholders are concerned that the environment functions as a reservoir for AMR, playing a critical role in spreading ARGs. Multiple factors drive the spread of antibiotic-resistant bacteria and their ARGs, which include the direct use of antimicrobial drugs in healthcare, agriculture, and livestock, as well as the release of antibiotic residues from various domestic sources into the environment [15]. Pesticides, heavy metals, medications, personal care items, and microbes linked to agriculture and human waste streams are all found in areas of pollution surrounding any human activity, and their spatial change and fold change determination is challenging [61]. To measure this potential spread and abundance of these antimicrobial resistance agents in the environment, integron integrase genes could be a possible proxy [61]. Integrons are common in bacteria, with about 15% of all of the sequenced bacterial genomes harboring these elements [62]. Integrons have the capacity to capture exogenous genes and express them, and the gene cassettes incorporated mostly confer resistance to AMA [57]. This function makes class 1 a promising proxy for measuring anthropogenic inputs and ARG abundance in the environment [63]. The integron integrase *Intl* gene is linked to the genes conferring resistance to antibiotics, disinfectants, and heavy metals, its presence in a wide range of environments, and pathogenic and non-pathogenic bacteria [61,64]. Furthermore, integrons the ability to transfer resistance genes among bacteria rapidly, through horizontal gene transfer and can be found in a wide range of xenogenetic elements [65]. Thus, the anthropogenic inputs and the resulting environmental pollution can be monitored through the integrons and can be helpful in mitigating this pollution.

### 4.3. AMR Dissemination in Wastewater

In the context of the ongoing global antimicrobial resistance crisis, it is essential to understand the interplay between clinical settings and environmental factors and to identify the primary drivers of AMR gene dissemination [19]. Studies have demonstrated that effluents from wastewater treatment plants (WWTPs) play a significant role in the spread of AMR with in sediment communities, both phenotypically and genotypically, and are a major driver of AMR into the aquatic ecosystem [19]. The highest diversity of ARGs has been found in WWTPs, which are significant entry points for pathogens and ARGs into downstream aquatic environments. WWTPs are also significant repositories of antibiotic resistance [66]. There are studies linking the environment and human resistome, and shared gene cassettes have been observed. This hypothesis is supported by the isolation of genetic resistance determinants from both human and environmental bacteria, demonstrating that the same AMR genes and associated elements were present in both [67,68]. This evidence reinforces the idea that environmental AMR is connected to clinical AMR; however, WWTP effluent increases the prevalence of AMR genes and phenotypes in the riverine system [67,69]. The enrichment of resistance to sulfonamides, aminoglycosides, and disinfectants is often linked to integrons, and has been observed from influent to effluent. This finding aligns with other studies that have reported a relative increase in ARGs following wastewater treatment, including those conferring resistance to macrolides, beta-lactams, aminoglycosides, sulfonamides, and vancomycin [70]. Some studies suggest that ARGs can be removed and their abundance can be decreased up to 90%; however, some resistance genes are still unable to be removed, such as *tet34* (tetracycline resistance), *bla1* (beta-lactam resistance), *vatC* (*MLSB* resistance), *vanTC* (vancomycin resistance), *bacA* (bacitracin resistance), *mexE*, *ttgA*, and *mtrC* (multidrug resistance) genes [71].

Effluents from WWTPs significantly drive large-scale shifts in the AMR phenotypes and genotypes of bacterial communities in sediments by introducing human-associated bacteria, thereby altering the resistomes of aquatic environments. The presence of class 1 integrons in both human and environmental bacteria indicates that bacteria from different environments may share mobile gene cassettes. Consequently, the emergence of AMR in riverine ecosystems poses a clinical concern, as it may facilitate the development of novel AMR gene cassettes in clinically relevant bacteria [72,73]. Additionally, the compositions of class 1 integrons in urban water systems and WWTPs appear to be similar in both quality and quantity [74], indicating that the composition of WWTP effluents mirrors that of urban wastewater [75]. During the treatment process, it is believed that 90% of integrons are removed, however, the normalized copy numbers of class 1 and class 2 integrons remain unchanged. Sludge treatment reduces the overall bacterial population, but does not significantly impact those that harbor integrons [76,77]. It has been observed that treatment reduces the diversity of gene cassette arrays; however, the most frequently detected gene cassettes confer resistance to quaternary ammonium compounds (QACs) and aminoglycosides. This highlights the need for new regulations to limit the use of QACs and improve waste water treatment practices.

### 4.4. Hospital Waste Water Effluent Impact on the Dissemination of Class 1 Integrons and AMR

The hospital environment plays a significant role in the spread of AMR due to the significant use of antibiotics and the discharge of treated and untreated effluents into surrounding ecosystems. Hospital effluent contains a large copy number of integrons, which are associated with antibiotic resistance, with approximately half of these integrons being class 1 integrons. The abundance of integrons was found to be influenced by working and non-working days, indicating a notable difference in their prevalence based on the day of operation. Additionally, the proportion of class 1 integrons containing at least one resistance gene was higher compared to that of other samples [78,79]. This clearly indicates that hospital settings play a significant role in the spread of antibiotic resistance, with most gene cassettes found in hospital effluent being linked to antibiotic resistance [80,81]. Gene cassettes conferring resistance to aminoglycosides, such as *aadA* and *aadB*, located on class 1 integrons, are prevalent in hospital effluent. The excessive use of specific antibiotics, including, amikacin, gentamicin, and tobramycin, in hospitals has contributed to the emergence of resistance, and this trend was confirmed by the French Public Health institute in 2010 [19]. However, the use of aminoglycosides is 11 times higher than that of other antibiotics, and their resistance is more prevalent in the hospital settings [82,83]. Hospitals are major consumers of QACs [84], and class 1 integrons play a significant role in the dissemination of QAC resistance across various environments [85]. The frequent use of disinfectants, surfactants, and other classes of antimicrobial agents may be the primary cause of AMR. It is essential to explore alternative strategies to reduce the use of these detergents and prevent their discharge into municipal drainage systems.

### 4.5. Fertilization and Increase in Environmental AMR and Integrons

Manure has been identified as a hot spot for bacterial communities that harbor antimicrobial-resistance genes associated with mobile genetic elements (MGEs) [19]. When manure is applied to soil, it introduces AMA, their metabolites, and bacteria carrying ARGs into the environment [86]. Additionally, soil naturally serves as a reservoir for ARGs, containing a diverse array of both known and unknown antimicrobial determinants [87,88]. The addition of AMA can exert selective pressure, potentially reducing the resilience of the soil bacterial community when manure is applied, due to the presence of certain AMA [19]. A wide host range of AMA and ARGs may increase the likelihood that commensal bacteria and human pathogens will acquire AMR through MGEs. This is similar to how plasmids enable HGT between related species [89,90]. Various soil amendments have impacted the abundance of soil ARGs and their subsequent dissemination in different environments, as listed in Table 2. Aminoglycosides, aminoglycoside, beta-lactamase, fluoroquinolone, quinolone, florfenicol, chloarmphenicol, MLSB, multidrug, sulfonamide, tetracycline, and vancomycin resistance genes, and MGEs are among the main families of antibiotic abundance that have been increased with the application of manure to a field, and, among them, the aminoglycosides are the most abundant [91]. The application of manure increased the abundance of ARGs by 116% in comparison to chemical fertilization, while the bio-organic fertilizers reduced this abundance by 31% [91]. In the soil treated with biogas slurry, the relative abundances of the majority of ARGs (*ereA*, *ereF*, *mefA*, *sul1*, *sul2*, *tetG*, and *tetO*) declined with time, but they were still much greater in the 5-year-treated soil than in the control soil; in addition, the *Intl* gene copy number was significantly higher in the soil treated with slurry, and this abundance increased with the time period [92]. In another study, manure application introduced 23% of new ARGs to the soil, and this number increased over time; moreover, the main classes of these ARGs included aminoglycoside, beta-lactamase, fluoroquinolone, quinolone, florfenicol, chloarmphenicol, MLSB, multidrug, sulfonamide, tetracycline, and vancomycin [93]. Another study suggested that 114 new ARGS were amplified from manure-treated soil, and, with up to 0.23 copies of the 16S rRNA gene and 81 distinct ARGs, the relative abundance and measurable amounts of ARGs were significantly boosted by the application of manure [94]. This change in ARGS and MGEs, specifically in integrons, was directly influenced by manure application, which led to the increased abundance in the AMA [95]. In the soil environment, a community of soil bacteria exists, and the ARGs are most likely transferred to these soil bacteria through horizontal gene transfer. This process is further enhanced by the introduction of manure to the soil [19]. Factors influencing the dissipation rate of ARGs include the transfer of ARGs to host bacteria via horizontal and vertical transmission, the transport of extracellular DNA containing ARGs, the attachment of ARGs to soil particles or organic matter, the degradation of extracellular ARGs, and the decline of bacterial hosts [89].

The addition of AMA to soil leads to their absorption by plants via passive uptake and water transport [103,104]. However, limited information is available regarding the interactions between AMA concentration in manure and soil, the chemical characteristics of AMA, crop characteristics, the different plant growth stages, and plant physiology in relation to AMA uptake. It has been observed that both treated and lake waters contain ARGs [19]. This suggests a possibility that fresh fruit may become contaminated with antibiotic-resistant bacteria (ARB) and ARG, due to the use of irrigation water from these sources for plant cultivation. Irrigation water is a significant source of bacterial contamination and plays an essential role in the contamination of vegetables during the pre-harvest phase [105,106]. As research on pathogens and food-borne illnesses progresses, more studies are identifying antibiotic-resistant bacteria on fruits and vegetables [107,108]. However, limited information is available regarding the correlation between the quantity and distribution of the relevant bacteria and ARGs on plant products, irrigation water, and manure containing ARGs and ARB. AMA are used less frequently in plant production compared to human and animal health systems, both from quantitative and qualitative perspectives. The primary method of AMA use in plant production involves spraying, which can lead to the contamination of soil and water resources [109]. The agriculture system plays a major role in the spread of integrons and AMR. Further studies are required in order to establish the connection between AMR and agriculture and to explore its role in the undiscovered reservoirs of gene cassettes in these environments.

### 4.6. Integrons in Marine and Freshwater Environments

Freshwater and marine environments impacted by human activities play a significant role in the spread of AMR, as highlighted by the One Health framework. These ecosystems can facilitate the spread of antibiotic-resistant bacteria, and act as reservoirs of resistance genes. Human-altered waters, in particular, may harbor ARGs, further contributing to the dissemination of AMR [110]. Many studies have focused on clinical sites, while fewer have investigated environmental sites. Some studies have examined the presence of *Intl1* sequences in soil [111,112], poultry litters [112], heavy-metal-contaminated mine sites, deep-sea sediments and polluted deep-sea environments [113], submarine gas-hydrate-bearing cores [114], and different terrestrial, deep-sea environments [115,116]. Research from freshwater reservoirs has identified approximately 322 distinct *Intl1* sequences groups, suggesting significant variation among *Intl1* genes and indicating that environmental factors influence the composition and evolution of these genes. This variation highlights how *Intl1* sequences adapt to specific environmental conditions [111,117].

The CL1 integron is used as a proxy for the pollution and dissemination of AMR in various environments, including the fresh and marine water bodies [15]. The study on fresh water bodies revealed an abundance of CL1 concentration that ranged from 4.22 × 10^−6^ to 4.08 × 10^−4^ gene copies/16S rRNA gene copy, and from 2.06 × 10^−5^ to 1.38 × 10^−2^ gene copies/bacterial cell; in addition, its abundance was higher in riverine water in comparison to spring and glaciers, which confirms that the anthropogenic impacts are higher in riverine systems and the CL1 abundance increases with this input [118]. The abundance of class 1 integrons is affected by seasonal changes, and their abundance increased in the winter season compared with that observed in the summer. This change may be linked to the influx of water flow [119]. A total of 24 different class 1 integrons associated with ARGs are detected, which are predicted to encode resistance to a wide range of antimicrobial classes, including aminoglycosides, beta-lactams, chloramphenicol, rifampicin, trimethoprim, and quaternary ammonium compounds [118,120]. Aminoglycosides and beta-lactamase resistance genes were the most frequently detected among the ARGs, which aligns with expectations, as this group is known for its diversity and prevalence in aquatic environments, as shown by metagenomic studies [121].

Marine habitats are exposed to various contaminants originating from both land and sea sources. Land-based contaminants primarily stem from industrial activities, urban wastewater, and agricultural practices, while sea-based pollutants are mainly attributed to crude oil spills from offshore drilling and shipping operations. Among these, salinity stands out as the key environmental factor shaping the composition of microbial communities [122,123]. In the marine environment, particularly along the shore, ARGs are common. ARGs have been shown to be highly abundant in the Red Sea, a significant maritime transportation route. In particular, ARGs *qnrS*, *aacC2*, *ermC*, and *blaTEM-1* are common [124]. The majority of maricultural sand samples include antibiotics and related ARGs, with sulfanilamide resistance genes being particularly common, according to a study of 11 typical maricultural regions along the Chinese coastline [125,126]. Another study on antibiotic resistance in fish raised in mariculture cage-culture systems revealed that *sul1*, *tetB*, and *ermB* genes were the most prevalent, and the identified ARGs were associated with opportunistic pathogens [127,128]. Likewise, a global metagenomic analysis of ARGs in various aquatic environments indicated that coastal seawater samples exhibited a higher relative abundance of ARGs compared to samples from the deep ocean and Antarctic regions [121]. The gene cassettes found in these sites are predominantly transcribed into hypothetical proteins with general functions, accounting for approximately 22% of the sequences [129,130]. Only a few of these sequences display known patterns or domains, although other studies have identified antibiotic-resistant gene cassettes as well [131,132]. Despite substantial efforts in sequencing large numbers of gene cassettes, rarefaction curves often reach saturation, indicating that a wide range of genetic diversity exists outside of clinical environments [113,115].

This approach highlights the significant large-scale sequencing of gene cassettes from nonclinical environments. Some of the gene cassettes in these environments exhibit homologies with those found in previously studied contaminated environments [132,133]. Interestingly, shared gene cassettes between freshwater environments and marine environments were identified, suggesting that their distributions occur more on a global scale due to anthropogenic input. Comparative studies of gene cassettes in relation to nearby environments indicate a co-assorting group of genes. It is suggested that the diversity of gene cassettes is affected by environmental pressure and contamination. Anthropogenic inputs are a major contributor to the dissemination of integrons and AMR in the environment.

## 5. Conclusions and Future Perspective

Antimicrobial resistance is a critical global health concern, influenced by both environmental and clinical factors. Among these, integrons, particularly class 1 integrons (CL1), play a crucial role in the horizontal spread of ARGs across bacterial populations. These MGEs are found in bacteria residing in diverse ecosystems that facilitate the spread of AMR, including freshwater systems, agricultural fields, WWTP, hospital settings, and marine environments. Anthropogenic activities, such as the overuse of antibiotics in healthcare, agriculture, and livestock production, exert selective pressure on bacterial communities, driving the acquisition and propagation of resistance genes. Major vectors for the spread of AMR include the use of contaminated irrigation water, effluents from WWTPs, manure application in agriculture, and wastewater treatment plant effluents from hospitals. The global environmental reservoirs of resistant bacteria and their associated genetic determinants, including integrons, can be transferred between ecosystems, exacerbating the global burden of AMR. The presence of integrons in diverse environments underlines the complexity and connectivity of the AMR problem. Integrons can be a good proxy for monitoring pollution and the dissemination of resistance, and they may be a tool that could track the circulation of ARGs between environmental and clinical settings. Marine and freshwater ecosystems can, in this respect, be considered hotspots for AMR, where human activities have left a mark, with plenty of evidence pointing toward the global spread of resistance.

The evidence gathered from clinical settings, hospital discharge, and agricultural runoff highlights the pressing need for thorough strategies to reduce the environmental spread of AMR. These strategies should encompass improved wastewater treatment, responsible antibiotic usage in agriculture, and enhanced waste management practices to prevent the release of resistance genes into the environment. Given the interconnected nature of human, animal, and environmental health, a coordinated One Health approach is essential to address the AMR crisis on a global level.

### Future Perspectives

Future research should address the development of integrated systems for monitoring AMR both at clinical and at environmental levels. The use of such molecular tools as meta-genomics, whole-genome sequencing, and real-time monitoring of integron abundance can generate a lot of valuable data on the spread of AMR. In this regard, the study of CL1 integrons as markers for environmental pollution and AMR dissemination may be a good approach to explain how resistance genes move across various types of ecosystems. Agriculture, especially with respect to manure and fertilizer use, needs to be more closely considered with regard to the dissemination of AMR. Future studies may wish to consider the dynamics of antibiotic resistance gene transfer in manure-amended soils and the implications for crop contamination, among others. A lot of research is needed with respect to the fate of ARGs in soil and their persistence in the environment after manure application. Besides that, other fertilization practices, such as bioorganic and biogas slurry treatments, should be further explored for potentially lower burdens of AMR in agroecosystems.

WWTPs are an important means of reducing the dissemination of AMR; however, the treatments applied up until now are quite ineffective in the complete removal of ARGs. Future developments must be created in order to increase the effectiveness of WWTPs, in particular on integrons and cassette genes, to further reduce their impact. The development of new disinfection techniques could significantly reduce the levels of antibiotic-resistant bacteria and resistome in effluents, such as through advanced oxidation processes or membrane filtration. The control of AMR requires a One Health approach, since human, animal, and environmental health are intricately linked. This means that future strategies need to be cross-sectoral, with surveillance and the implementation of AMR control measures at the human–animal–environment interface. This calls for responsible use of antibiotics both in healthcare and agriculture, better hygiene and sanitation practices in hospitals, and enhanced biosecurity measures in livestock farming.

Increasing public awareness of the contribution and role that AMR, environmental impact, and poor wastewater management play in spreading resistance is imperative. Policymakers have to give more prominence to AMR in environmental health agendas, ensuring that regulations concerning the use of antibiotics in agriculture and animal husbandry are enforced and environmental contamination is monitored more rigorously. Human campaigns on the avoidance of unnecessary antibiotic use, along with proper pharmaceutical disposal practices, are complementary measures that are needed to ensure that the effectiveness of such strategies against AMR in the long run will be matched. AMR is a global problem, therefore, future work must involve international collaboration in sharing data, research findings, and best practices in handling resistance across borders. This will be important in building global surveillance networks that track AMR in real time, especially in low- and middle-income countries where antibiotic use and environmental pollution are rising. In the future, new gene cassettes of AMR are likely to be uncovered from nonclinical settings, including soil, freshwater, and marine ecosystems. Extensive sequencing would be required for identifying and characterizing the previously unknown resistance mechanisms circulating in the environment, with the aim of developing new diagnostic tools and therapeutic strategies against emerging forms of resistance.

## 6. Conclusions

The global spread of AMR has become a multidimensional problem requiring coordination in efforts between the human health, animal health, and environmental sectors. The understanding of integrons’ role in AMR dissemination, the enhancement of our monitoring system and technologies for wastewater treatment, and good agricultural practices would be relevant steps toward mitigating the environmental spread of resistance. This could be achieved through the implementation of a One Health approach, coupled with research, innovatively and globally, to overcome the expanding crisis of AMR and secure public health for future generations.

## Figures and Tables

**Table 2:** Various soil amendment impacts on antibiotic resistance under different environments.

Fertilizer Types	Effects on AMR Genes	Percent Increase	Reference
Manure and bio-organic fertilizer application	Aminoglycosides, beta-lactamases, chloramphenicol, macrolide-lincosamide-streptograminB (MLSB), multidrug, sulfonamide, tetracycline, vancomycin resistance genes	116%	[90]
Mineral fertilizer (NH_4_NO_3_), cattle slurry, and cattle slurry digestate amendment	Tetracycline, sulfonamides, macrolides, integrase gene copy number increased	83%, 20%, 64%, 83%, log copies/gm soil	[96]
Composted manure	Aminoglycoside, bacitracin, chloramphenicol, sulfonamide, tetracycline, and multidrug resistance was present in higher abundances than the other resistance genes	24% increase in total abundance	[97]
Cattle slurry digestate	*TetA*, *blaCTX-M*, *blaOXA2*, *qnrS*, intI1, and intI2	104–105 copies/gm soil and (1.2 × 109 copies/gm soil)	[98,99]
Swine manure	ARGs (ermB, qnrS, acc(6′)-Ib, tetM, tetO, and tetQ) tetQ and tetW, and ermB and ermF	3.01 × 108 to 7.18 × 1014 copies/g	[100]
Manure applications	*CL1*, *QACs*, sulfonamide, tetracycline, and multidrug	109 copies/gm and 16–48% increase	[18]
Organic fertilizers and livestock and poultry manure	ARGs, including sul2, TetB-01, TetG-01, and TetM-01, TetK, and ermC	12–96%	[101]
Organic fertilizers	*IntI1*, *sul1*, and *tetM*, *blaTEM*, and *blaOXA-48*, *qnrS1*	20–100-fold increase change	[102]

## Data Availability

Data sharing is not applicable to this article as no datasets were generated or analyzed during the current study.
